# Acid-Sensing Ion Channel 1 Contributes to Weak Acid-Induced Migration of Human Malignant Glioma Cells

**DOI:** 10.3389/fphys.2021.734418

**Published:** 2021-09-07

**Authors:** Sareena Shah, Yuyang Chu, Victoria Cegielski, Xiang-Ping Chu

**Affiliations:** ^1^Department of Biomedical Sciences, School of Medicine, University of Missouri-Kansas City, Kansas City, MO, United States; ^2^Feinberg School of Medicine, Northwestern University, Chicago, IL, United States

**Keywords:** acid-sensing ion channel 1, human malignant glioma cells, migration, acidosis, patch-clamp recording

## Introduction

Glioblastoma multiform (GBM), also referred to as glioblastoma, is the most common malignant tumor in the brain, known for its resistance to therapeutic agents and poor prognosis (Shergalis et al., 2018). Recent studies have seen success of some target compounds in combating GBMs in laboratory settings; however, these compounds failed in clinical trials (Mandel et al., 2018). Multiple factors may have contributed to this drug failure in GBM treatment, including difficulty penetrating the blood-brain barrier (BBB), an immunosuppressive microenvironment, and complex intratumoral heterogeneity (Khaddour et al., 2020; Ou et al., 2020). Difficulty in treating GBMs has led to a 5-year survival rate of <7.2% in humans. This is the lowest long-term rate of malignant brain tumors (Ostrom et al., 2020). Current treatment options for patients with GBM include surgical resection and a combination of chemotherapy, radiotherapy, and immunotherapy (Carlsson et al., 2014; Suter et al., 2020; Wen et al., 2020; Medikonda et al., 2021). Surgical resection is the most effective way to increase short-term survival rate, with research demonstrating a higher 1-year survival rate in patients with at least a 90% surgical resection compared to those without one (Medikonda et al., 2021). Yet, the long-term survival rate remains the same, suggesting that tumor recurrence is very likely (Yu et al., 2021).

GBMs, like many other tumors, have an acidic microenvironment due to the tumor's high rate of metabolism and limited blood supply (Tian et al., 2017). Acidosis may result from an accumulation of acid or an increase of partial pressure of carbon dioxide in tissues, which can cause lactic acid build-up in the brain (Chesler, 2003). Excessive lactic acid in the brain can damage metabolic functionality, hindering recirculation and reoxygenation throughout the body (Siesjö, 1982; Siesj et al., 1993; Rehncrona, 2005). Studies have demonstrated that acidosis can activate a family of ligand-gated ion channels: acid-sensing ion channels (ASICs), which are widely expressed in neurons (Waldmann et al., 1997; Xiong et al., 2004; Krishtal, 2015). ASICs are formed through different combinations of subunits, either homotrimeric or heterotrimeric, constituting different electrophysiological and pharmacological properties (Gründer and Pusch, 2015; Vullo and Kellenberger, 2020). The activation of ASICs induces neuronal depolarization and generates action potentials mostly due to the influx of Na^+^ ions (Jiang et al., 2009; Boscardin et al., 2016). ASICs play critical roles in several physiological processes including synaptic plasticity, pain sensation, and fear conditioning (Wemmie et al., 2013; Huang et al., 2015; Uchitel et al., 2019; Storozhuka et al., 2021). They also contribute to several neurodegenerative disorders such as Parkinson's disease and multiple sclerosis (Chu and Xiong, 2012; Chu et al., 2014; Ortega-Ramírez et al., 2017). Additionally, ASICs are expressed in glial cells (Lin et al., 2010), particularly ASIC1, whose activation in glial cell lines has been linked to migration and proliferation of the GBMs (Berdiev et al., 2003; Bubien et al., 2004; Kapoor et al., 2009, 2011; Rooj et al., 2012). Glioma tumors aggressively destroy surrounding tissues while growing in the brain, displaying acidosis and resulting in pathophysiological consequences (Honasoge and Sontheimer, 2013). ASIC expression in glioma cells generates a Na^+^ current which contributes to its volume and migration (Rooj et al., 2012). Active conductance has been observed in gliomas due to the presence of ASICs and epithelial sodium channels, which can impact cell cycle progression (Kapoor et al., 2009). The impact of ASICs, particularly ASIC1, in glial cell lines is being further investigated in efforts to reduce glial cell migration, study the potential proliferation effects, and to understand how ASIC1 inhibitors can regulate apoptosis of glioma cells (Sun et al., 2013; Tian et al., 2017). ASIC1 gene expression in glioma cells can predict outcomes of carcinomas as they act as genomic biomarkers; therefore, finding more inhibitors to ASICs may provide potential therapeutic agents to inhibit glioma cell activity (Sun et al., 2013; Bychkov et al., 2020; Griffin et al., 2020).

## ASIC1 Contributes to Weak Acid-Induced Migration of Human Malignant Cells

A recent study from Dr. Xiong's laboratory published in the *American Journal of Cancer Research* described a combination of wet-lab techniques, including whole-cell patch-clamp recording, Western blotting, cell viability assay, wound-healing assay and trans-well migration assay, that were used to determine if the activation of ASIC1 by a weak acid (e.g., pH 7.0) had a correlation with the migration and proliferation of glial cell lines U87MG and A172 (Sheng et al., 2021). The study determined the expression of ASIC1 in glioblastoma cell lines, along with the induction of transient inward currents when the pH was dropped from 7.4 to 6.0, suggesting the existence of acid-activated currents in glioblastoma cell lines. When PcTx1, a selective ASIC1 inhibitor, was introduced, there was a decrease in acid-activated currents, indicating that ASIC1 was involved. The study also examined cell viability of A172 and U87MG cells when treated under different pH conditions (e.g., pH 7.4 and pH 7.0) to determine if weak acidosis could affect proliferation of the cells; however, they found that neither acidosis nor the addition of PcTx1 had an influence on cell viability of the two glioma cells, suggesting that weak acidosis has no effect on proliferation of human glioma cells. Using a wound healing assay, they investigated whether weak acid could promote migration of A172 and U87MG cells and found that weak acid did increase the migration rate by 26% in A172 cells and 67% in U87MG cells, respectively. When PcTx1 was introduced, it inhibited both glioma cells from migrating. Further investigation of this migration was conducted using a trans-well migration assay and revealed similar results. They also found that there was a heavier expression of the ASIC1 protein in U87MG cells compared to the expression in A172 cells, indicating that different cell lines of GBMs have different levels of ASIC1 expression. The study also introduced ASIC1-siRNA to U87MG cells, which silences ASIC1 expression, to better understand the influence of this protein on migration induced by treatment of weak acidosis. When compared to the control group, application of ASIC1-siRNA decreased the expression of the ASIC1 protein and did not affect the proliferation of U87MG cells, but it reduced the migration of U87MG cells under weak acidic conditions. Collectively, the results demonstrate that activation of ASIC1 by weak acidosis in glioma cells A172 and U87MG promotes migration of the cells but not proliferation, while PcTx1 inhibits migration of glioma cells. These findings suggest that ASIC1 could serve as a potential therapeutic target for GBMs.

## Perspective

Although there are several studies of ASICs in the pathophysiology of glioma cells (Berdiev et al., 2003; Bubien et al., 2004; Kapoor et al., 2009, 2011; Rooj et al., 2012), this study uniquely dives deeper into the connection between weak acidosis activated ASIC1 and its effect on the migration of glioma cells (Sheng et al., 2021). A recent study from Dr. Grunder's group reported that glioblastoma stem cell (GSC) lines (R8 and R54) express functional ASIC1 and ASIC3, and their data suggest that expression of ASICs is associated with an improved survival in GSC lines (Tian et al., 2017). This result is inconsistent with other studies (Kapoor et al., 2009; Sheng et al., 2021). One possibility might be due to different glioblastoma cell lines used in different studies, suggesting the complexity of GBM. Therefore, additional studies might be necessary to further delineate the precise role of ASIC1 in high-grade glioblastoma. This study solely focuses on glial cells U87MG and A172, so it would be beneficial to utilize the same wet-lab techniques for cultured human glioma cells directly from the patients and see if parallel correlations are found. It would also be interesting to test PcTx1 on cultured human glioma cells to see whether it has any effects on glioma growth during a small pH drop (pH 7.0). In most whole-cell recordings, ASICs deactivate fast in response to brief pH stimulation (Gründer and Pusch, 2015). Even a small decrease in pH leads to profound steady state desensitization of ASICs. Therefore, it is intriguing how migration of high-grade gliomas could be promoted via ASIC continuously subjected to a slightly acidic pH, and the exact mechanism should be explored in the near future. Interestingly, a recent study demonstrates that the deactivation of ASIC1a is steeply dependent on the pH, spanning nearly three orders of magnitude from extremely fast (<1 ms) at pH 8.0 to very slow (more than 300 ms) at pH 7.0 (MacLean and Jayaraman, 2017). In addition, like most of ligand-gated ion channels, the desensitization of ASIC is also pH-dependent, with much slower desensitization occurs in response to a smaller pH drop (e.g., to 7.0) than a bigger pH drop (e.g., to 6.0). Although the current amplitude might be small with a small pH drop (Sheng et al., 2021), the current may last for a long period of time. Another possibility is that, in intact cells where cellular components are not washed out like in whole-cell recordings, the kinetics of ASIC1a current could be completely different. Additionally, the study from Dr. Xiong's group (Sheng et al., 2021) focuses on glial cells U87MG and A172 being introduced to a weak acid, but it would be insightful to compare the migration of these glial cell lines when introduced to a larger pH drop (e.g., pH 6.0). Further investigation may determine the consequences of cell migration under permanent inhibition of ASIC1 in glioma cells. Several existing studies have used PcTx1 or Mambalgin-2 as an ASIC1 inhibitor (Kellenberger and Schild, 2015; Dibas et al., 2019). However, it is necessary to explore other therapeutic agents since PcTx1 and Mambalgin-2 cannot be used in clinical settings due to their inability to cross the BBB (Sun et al., 2013; Bychkov et al., 2020). Therefore, determining effective delivery pathways for ASIC1 inhibitors is needed for the clinical treatment of GBMs. From a more malignant standpoint, late-stage glioma cells express high levels of ASIC1a, which is not portrayed in normal astrocytes. Thus, learning more about how and why ASIC1 expression changes in higher-grade gliomas may be vital in determining optimal treatment plans for patients with gliomas (Sun et al., 2013; Wang et al., 2015).

## Author Contributions

All authors listed have made a substantial, direct and intellectual contribution to the work and approved it for publication.

## Funding

The work was supported by grant from American Heart Association (19AIREA34470007) to X-PC.

## Conflict of Interest

The authors declare that the research was conducted in the absence of any commercial or financial relationships that could be construed as a potential conflict of interest.

## Publisher's Note

All claims expressed in this article are solely those of the authors and do not necessarily represent those of their affiliated organizations, or those of the publisher, the editors and the reviewers. Any product that may be evaluated in this article, or claim that may be made by its manufacturer, is not guaranteed or endorsed by the publisher.
